# Deep transfer learning to quantify pleural effusion severity in chest X-rays

**DOI:** 10.1186/s12880-022-00827-0

**Published:** 2022-05-27

**Authors:** Tao Huang, Rui Yang, Longbin Shen, Aozi Feng, Li Li, Ningxia He, Shuna Li, Liying Huang, Jun Lyu

**Affiliations:** 1 Department of Clinical Research, The First Affiliated Hospital of Jinan University, Guangzhou, 510630 China; 2 Department of Rehabilitation Medicine, The First Affiliated Hospital of Jinan University, Guangzhou, 510630 China; 3 Guangdong Provincial Key Laboratory of Traditional Chinese Medicine Informatization, Guangzhou, Guangdong China

**Keywords:** Pleural effusion, Severity, Deep learning, X-rays, Chest radiographs, MIMIC-CXR

## Abstract

**Purpose:**

The detection of pleural effusion in chest radiography is crucial for doctors to make timely treatment decisions for patients with chronic obstructive pulmonary disease. We used the MIMIC-CXR database to develop a deep learning model to quantify pleural effusion severity in chest radiographs.

**Methods:**

The Medical Information Mart for Intensive Care Chest X-ray (MIMIC-CXR) dataset was divided into patients ‘with’ or ‘without’ chronic obstructive pulmonary disease (COPD). The label of pleural effusion severity was obtained from the extracted COPD radiology reports and classified into four categories: no effusion, small effusion, moderate effusion, and large effusion. A total of 200 datasets were randomly sampled to manually check each item and determine whether the tags are correct. A professional doctor re-tagged these items as a verification cohort without knowing their previous tags. The learning models include eight common network structures including Resnet, DenseNet, and GoogleNET. Three data processing methods (no sampling, downsampling, and upsampling) and two loss algorithms (focal loss and cross-entropy loss) were used for unbalanced data. The Neural Network Intelligence tool was applied to train the model. Receiver operating characteristic curves, Area under the curve, and confusion matrix were employed to evaluate the model results. Grad-CAM was used for model interpretation.

**Results:**

Among the 8533 patients, 15,620 chest X-rays with clearly marked pleural effusion severity were obtained (no effusion, 5685; small effusion, 4877; moderate effusion, 3657; and large effusion, 1401). The error rate of the manual check label was 6.5%, and the error rate of the doctor’s relabeling was 11.0%. The highest accuracy rate of the optimized model was 73.07. The micro-average AUCs of the testing and validation cohorts was 0.89 and 0.90, respectively, and their macro-average AUCs were 0.86 and 0.89, respectively. The AUC of the distinguishing results of each class and the other three classes were 0.95 and 0.94, 0.76 and 0.83, 0.85 and 0.83, and 0.87 and 0.93.

**Conclusion:**

The deep transfer learning model can grade the severity of pleural effusion.

## Introduction

Pleural effusion is a common clinical symptom characterized by pathological fluid accumulation in the pleural cavity [1, 2] and is related to more than 50 causes [3]. Congestive heart failure, pneumonia, pleural lung cancer, connective tissue disease, acute pancreatitis, and trauma may all cause an increase in pleural effusion [4, 5]. In the ICUs, the diagnosis of pleural effusion relies mostly on the anteroposterior chest radiograph obtained at the bedside while the patient is in the supine position [6]. Severe pleural effusion in critically ill patients may contribute to hypoxemia under mechanical ventilation [7] or lead to tamponade physiology [8]. Quantitatively assessing pleural effusion volume is essential to help identify critically ill patients for thoracentesis [9].

Deep learning is a type of artificial intelligence that allows computers to learn without being explicitly programmed for a given task. More and more applications in medical imaging. Using deep transfer learning algorithms can build efficient, objective, and accurate disease diagnosis and identification models [10–12]. Diamant et al. [13] used transfer learning for pathological classification of chest radiographs and achieved high AUC results, demonstrating the strength and robustness of CNN extraction features. Niehues et al. [14] developed and evaluated a deep learning model for identifying clinically relevant abnormalities in bedside CXR, demonstrating that a bedside CXR-specific built based on a deep learning model showed similar performance to radiologists. However, these existing works all detect multiple different diseases, such as the presence of cardiac congestion, pleural effusion, air cavity opacity, pneumothorax and other diseases, ignoring the judgment of the severity of the disease. The automated and accurate detection of pleural effusion severity is conducive to clinicians’ rapid and reliable diagnosis of patient condition and relieves radiologists’ work pressure. This study focuses on patients with COPD, but the quantification of pleural effusion on chest radiographs is useful throughout clinical medicine.

Large-scale and general-purpose medical datasets are the catalyst for deep learning [15, 16]. The release of X-ray chest radiograph datasets [17–19] has greatly promoted the realization of deep learning-based chest disease recognition [20–22] and lesion detection on chest radiographs [3]. However, existing deep learning classification methods have not yet been verified on the multi-layered recognition of pleural effusion. MIMIC-CXR [17] is a large publicly available X-ray film data set with free-text radiology reports, and MIMIC-CXR-JPG [23] converts its DICOM format chest film to JPG format. This work extracts the severity label of pleural effusion from radiology reports and develops a universal and clinically significant deep learning recognition model that automatically and accurately judges the severity of pleural effusion on chest radiographs.

## Methods

### Data source

This historical cohort study used data from the free and open-access medical imaging database (MIMIC-CXR [17] and MIMIC-CXR-JPG [23] database version 2.0.0), which contains 227,835 data from 2011 to 2016 at the Beth Israel Deaconess Medical Center in Boston, Massachusetts. The dataset was de-identified to meet the requirements of the US Health Insurance Portability and Accountability Act of Safe Harbor of 1996 [24]. Protected health information was also deleted. The corresponding access agreement was signed, the dataset was downloaded, and the preprocessing was performed using data mining methods [25]. This research was conducted in accordance with the Declaration of Helsinki [26] describing the ethical principles of medical research involving human subjects.

### Data extraction and utilization

**Fig. 1:**
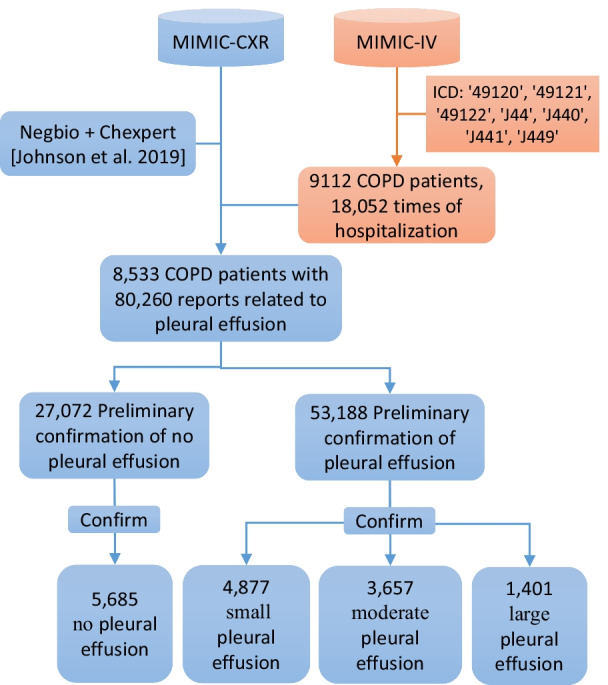
Data flow diagram

As shown in Fig. 1, we identified 9112 COPD patients from MIMIC-IV based on ICD 3 and ICD 4 diagnosis. Combined with imaging reports in MIMIC-CXR that were clearly expressed as with or without pleural effusion after processing by Negbi [27] and Chexpert [18]. According to the ID correspondence, 80,260 imaging reports of 8533 COPD patients were obtained. Note that each patient may have multiple admissions due to the disease, and multiple X-rays may be taken per admission to track patient status. We retrieved image reports from it using explicit text representations (as shown in Table 1), which were grouped into four attributes: No, Small, Moderate, Large of pleural effusion. Finally, data of 8533 patients diagnosed with COPD (mean age 65.40 years, 47.47% women) were extracted. Each patient may have multiple hospital admissions or multiple examinations. The entire dataset was split according to the patient’s independent ID, and randomly divided into training cohort, validation cohort and test cohort according to the ratio of 6:1:3. This ensures that the same patient will not be split into different sets. Since the splitting process is completely randomized and only 1–2 frontal lobe x-rays are taken per patient, bias in the dataset can be avoided. The NNI tool [28] in this paper provided the results of the verification cohort, and the other evaluations were completed on the testing cohort to ensure the reliability of the model.

**Table 1:** Explicit text representation for precise extraction of labels

No pleural effusion	Small pleural effusion
No pleural effusion	Tiny bilateral pleural effusions
Effusions have resolved	Tiny left pleural effusions
Without vascular congestion or pleural effusion	Tiny right pleural effusions
No vascular congestion, pleural effusion	Small bilateral pleural effusions
No pneumothorax, effusion	Small left pleural effusions
No appreciable pleural effusion	Small right pleural effusions
	Pleural effusions are small
	Small right fissural pleural effusion
	Small pleural effusion
	Tiny bilateral effusion
	Tiny left effusion
	Tiny right effusion

Moderate pleural effusion	Large pleural effusion
Moderate left pleural effusion	Large pleural effusion
Moderate pleural effusion	Large left pleural effusion
Moderate right pleural effusion	Large right pleural effusion
Moderate effusion	Large effusion
Moderate left effusion	Large left effusion
Moderate right effusion	Large right effusion
	Severe pleural effusion
	Severe left pleural effusion
	Severe right pleural effusion
	Large amount of loculated pleural fluid
	Large amount of pleural fluid
	Large amount of fluid

### Label extraction and validation

From the structured labels of MIMIC-CXR-JPG [23], we identified a batch of X-rays images that were clearly diagnosed as ‘with’ or ‘without’ pleural effusion. Referring to the method of Wang et al. [29], four severity level labels were obtained according to keywords matching rule and marked as follows: 0, no pleural effusion; 1, small pleural effusion; 2, moderate pleural effusion; and 3, large pleural effusion.

In order to verify the validity of the labels extracted from the radiology report, we randomly selected 200 X-ray images. A radiologist checks the radiology reports one by one, obtains artificial labels, and compares them with labels obtained based on matching rules to verify the accuracy of the rule labels.

In addition, three senior attending physicians marked these 200 X-rays as an additional verification cohort. It is used to verify the accuracy and reliability of the model’s prediction results. The three physicians did not know the labels we got from the radiology report in advance, but only marked which of the four severity levels for the image.

### Model development

Judging disease severity in medical images is a multi-classification problem of unbalanced distribution data. This paper had tried eight common deep learning network structures (DenseNet [30], DenseNet121 [30], GoogLeNet [31], Inception_V3 [32], MobileNetV2 [33], ResNet18 [34], ResNet50 [34], and AlexNet [35]) to build our model, and all of the structures have been proven effective in classification results on other datasets. We only fixed the parameters of the first two layers of the model. And the last output layer of the structure was modified to make it available for four classifications. In general, the last linear classification layer of the original network structure outputs a probability value for being a positive class in a two-class classification problem. In the four-class classification problem, the output was increased to four probability values representing the probability that the current data belongs to one of the four categories. The position with the largest probability value was designated as the classification result.

Three data processing methods (no processing, down sampling, and up sampling) and two loss algorithms (cross entropy and focal loss [36]) were used for category imbalance data. Five optimizers (SGD [37], Adadelta [38], Adagrad, Adam [39], and Adamax [39]) and three learning rates (0.005, 0.001, and 0.0005) were also added. During training, random image translation, rotation and normalization for data enhancement were performed to improve the robustness of the model. The Neural Network Intelligence (NNI) [28] tool was employed to optimize the model parameters.

### Statistical analysis

For the labeled dataset, the model with the best performance was selected after adjusting the parameters of the NNI [28] for further verification. For the testing cohort and verification cohort, the receiver operating characteristic curve (ROC) was drawn. We calculated the AUC classification results of a certain category and the other three categories, also each pairwise comparison. And drawn the corresponding confusion matrix. Grad-CAM [40] was used to generate a heat map to visualize the information area in the radiograph for the assessment of pleural effusion severity to explain the model prediction.

## Results

### Data analysis

**Fig. 2:**
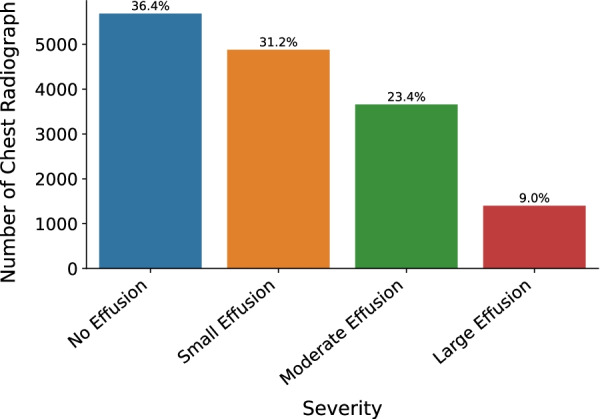
The number of patients of varying severity. The more severe the illness, the less data volume

**Fig. 3:**
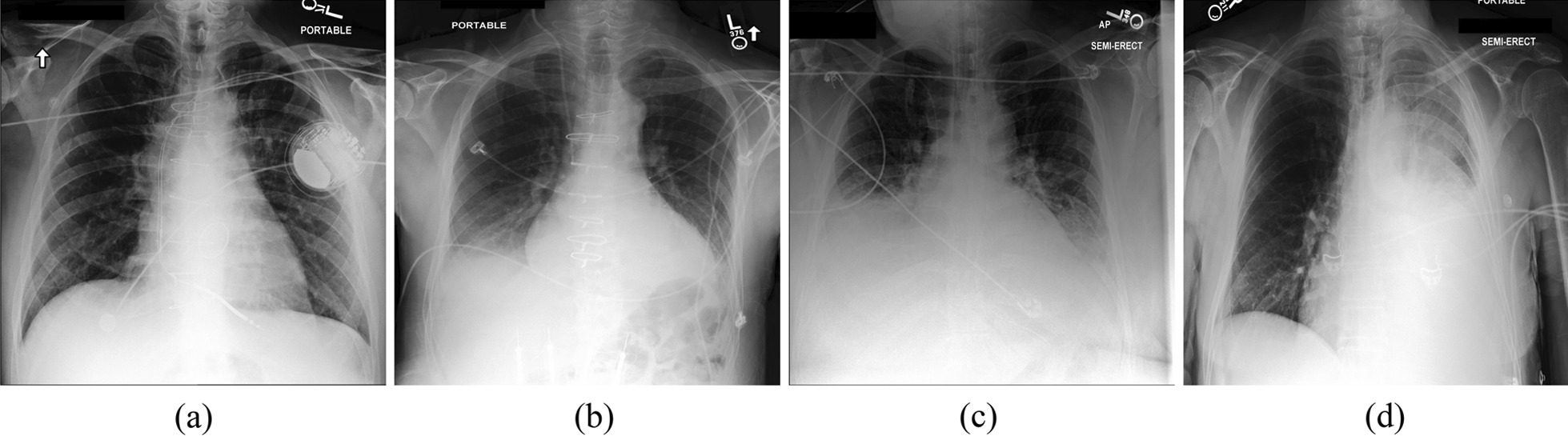
Examples of X-rays of pleural effusions of varying severity. **a** No effusion; **b** Small effusion; **c** Moderate effusion; **d** Large effusion

The pleural effusion data group had 432 more male patients than females (male: 4477, 52.53% and female: 4045, 47.47%) with an average age of 72 years (upper and lower quartile [55, 79]). The youngest age is 18 years old and the biggest is 91 years old. Each patient (8522 patients) had one or more radiographic studies and obtained 15,620 positive X-rays image data. As shown in Fig. 2, these data were divided into four groups: 5685 (36.4%) had no pleural effusion, 4877 (31.2%) had small pleural effusion, 3657 (23.4%) had moderate pleural effusion, and 1401 (9.0%) had severe pleural effusion. Figure 3 shows the randomly selected samples of pleural effusion with different severities.

### Label verification

**Fig. 4:**
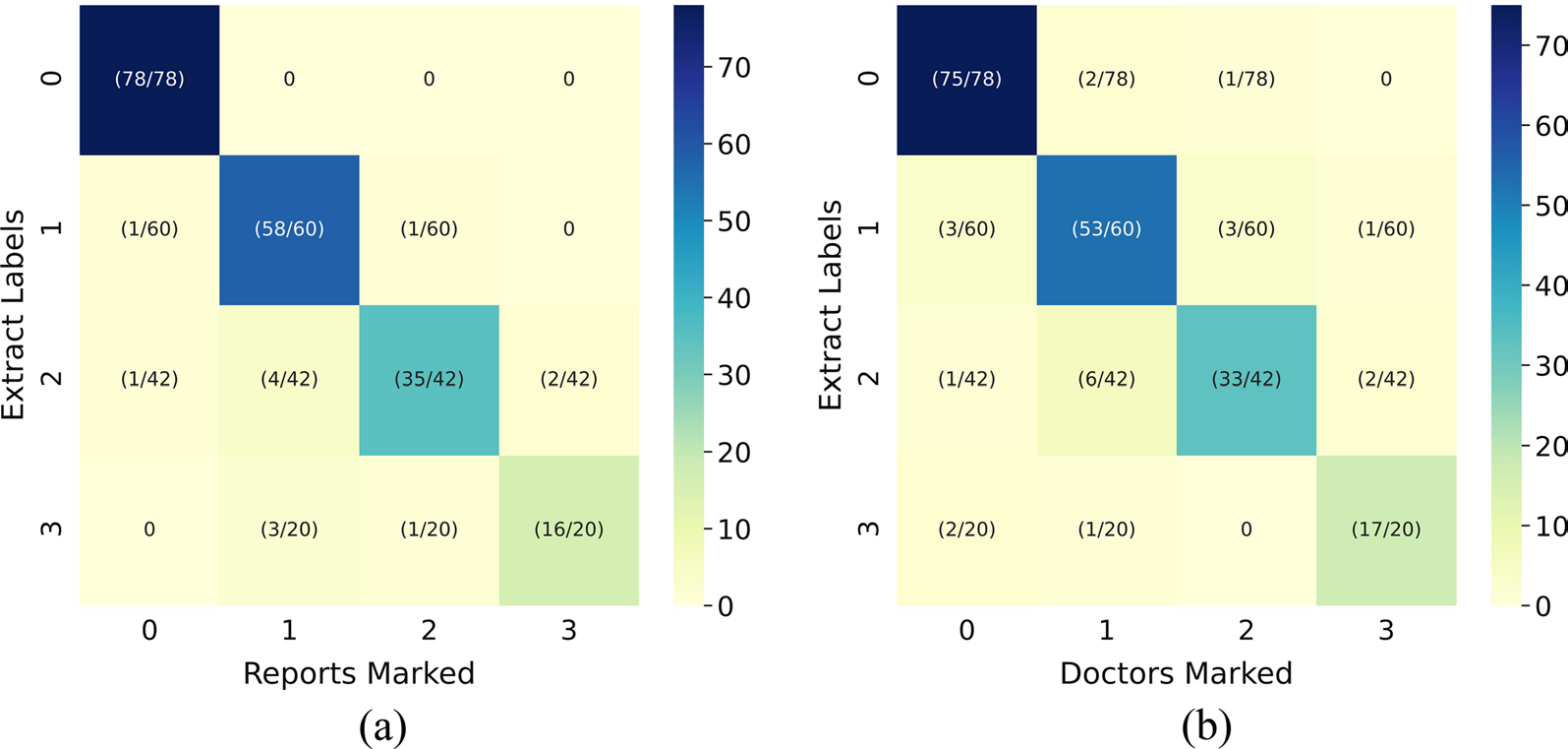
Comparison of 200 annotated results. **a** The results are extracted from the report based on the rules and compared with the results of the radiologist's inspection report item by item. **b** Comparison of the results extracted from the report based on rules and the results of the X-rays marked by the attending physician item by item

The labels extracted from the radiology report based on a fixed rule search were compared with those obtained from the radiologist’s itemized report inspection, and the deviation rate was 6.5%. As shown in Fig. 4a shown, no deviation was marked as no pleural effusion (label 0). Among the X-ray chest radiographs marked as small pleural effusion after regular retrieval, one was actually described as no pleural effusion after manual inspection, and another was actually described as moderate pleural effusion. The X-rays of moderate pleural effusion had many deviations in labeling; four actually showed small pleural effusion after examination, two were severe pleural effusion, and one was no pleural effusion. Among the X-ray films of severe effusion, three were actually small effusion, and one was moderate effusion.

The labels extracted based on the fixed rule search were compared with those obtained by the doctor’s label, and the deviation rate was 11.0%. The specific comparison is shown in Fig. 4b. The main deviation appeared in the judgment between small and moderate effusion.

### Model optimization

**Fig. 5:**
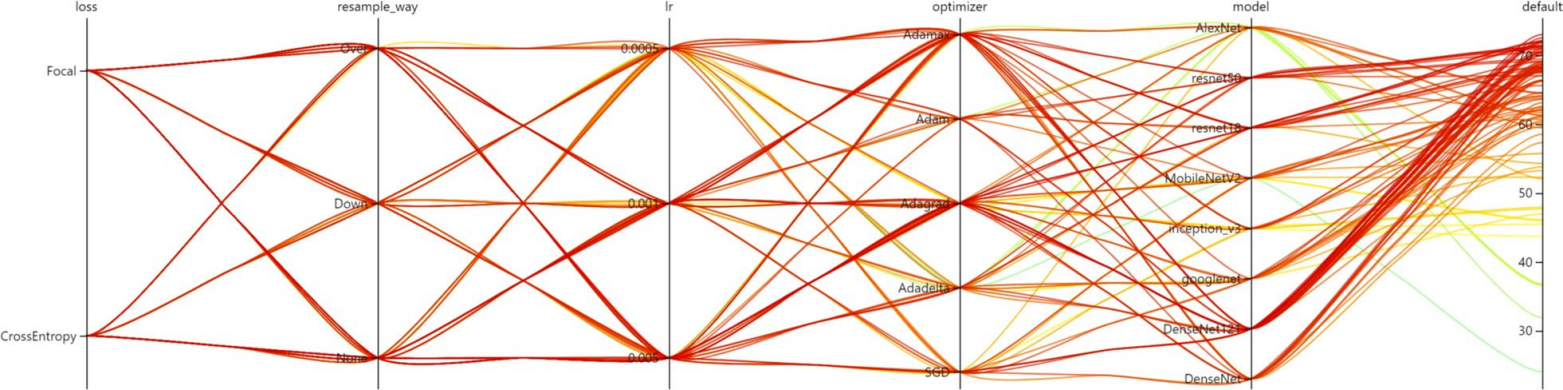
Hyperparameter optimization using an automated machine learning toolkit—NNI. Each line represents a trial, and the green to red color represents its accuracy from low to high

NNI optimization results revealed that the best model is Densenet121 with corresponding accuracy rate of 73.07%. The data were not sampled. Adagrad optimizer with a learning rate of 0.005 and focal loss function was used to calculate the optimal network parameters. The top 10 results of NNI optimization accuracy are shown in Table 2. Detailed parameters and optimized accuracy were also presented. The result of the parameter optimization line graph is shown in Fig. 5.

**Table 2:** Top 10 accuracy parameters, models and results optimized by NNI

	Data Sample	Loss	LR	Optimizer	Model	Accuracy (%)
1	None	Focal	0.005	Adagrad	DenseNet121	73.07
2	None	CrossEntropy	0.005	Adagrad	DenseNet121	72.56
3	None	CrossEntropy	0.001	Adamax	resnet18	72.19
4	Over sample	Focal	0.005	Adagrad	DenseNet121	72.04
5	None	CrossEntropy	0.005	Adagrad	resnet18	71.98
6	None	CrossEntropy	0.005	SGD	DenseNet121	71.94
7	None	Focal	0.005	Adagrad	resnet18	71.83
8	None	CrossEntropy	0.001	Adagrad	resnet18	71.51
9	None	CrossEntropy	0.005	Adagrad	resnet50	71.34
10	Over sample	CrossEntropy	0.001	Adagrad	resnet50	71.30

### ROC analysis

**Fig. 6:**
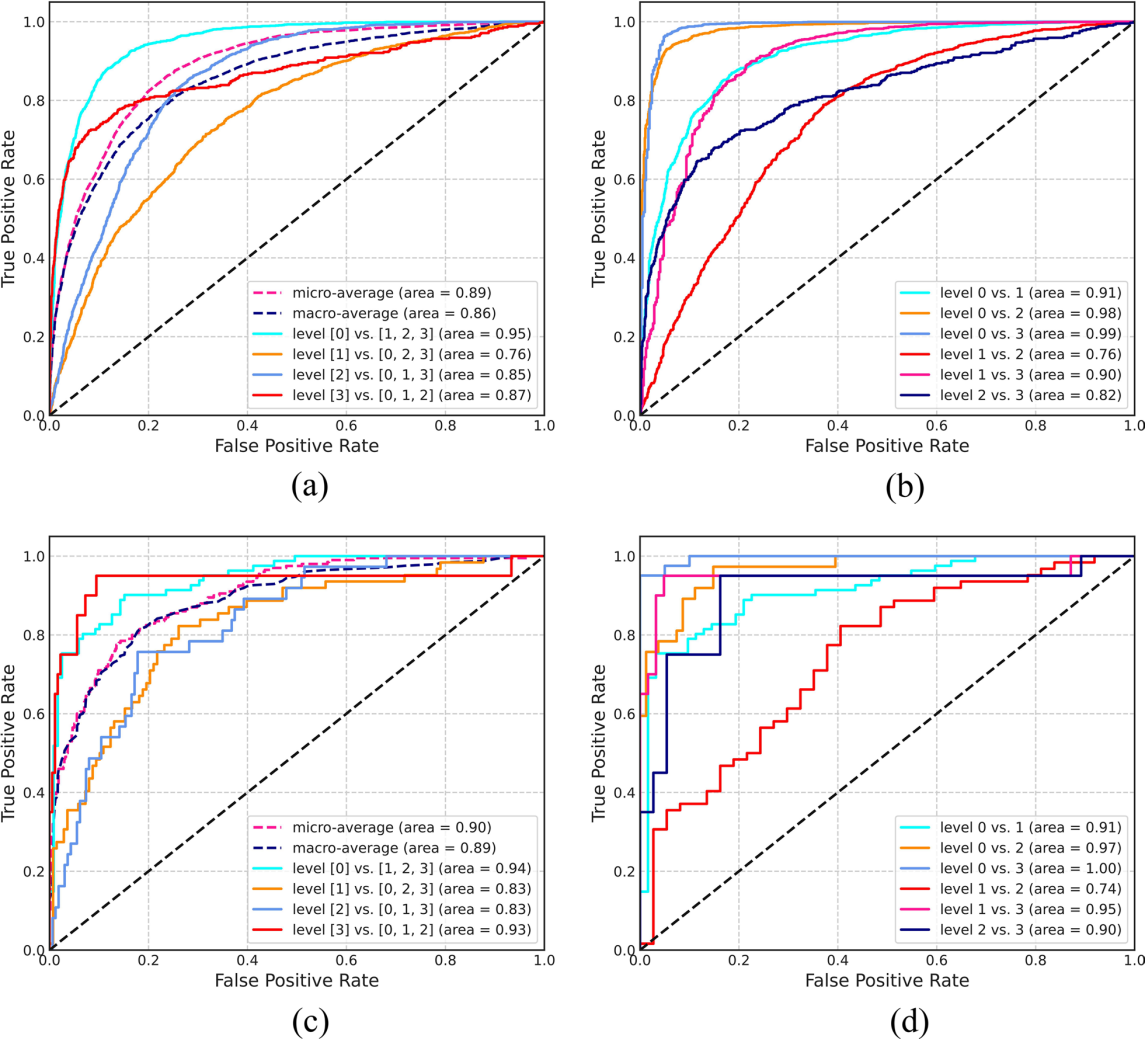
Receiver operating characteristic (ROC) curves of the testing cohort and validation cohort. **a** ROC curve of the single category compared with the other three categories of the testing cohort. **b** ROC curves for six pairwise comparisons of the testing cohort. **c** ROC curve of the single category compared with the other three categories of the validation cohort. **d** ROC curves for six pairwise comparisons of the validation cohort

Figure 6a displays the ROC curve of the testing cohort for each category compared with that of the other three groups. Clear distinction was achieved between patients with or without pleural effusion with an AUC of 0.95. Small pleural effusion was poorly distinguished from other severity levels with an AUC of 0.76. The micro average was close to the macro average (0.89 and 0.86). Figure 6b shows the distinction between any two categories of the testing cohort. The adjacent group exhibited a relatively lower AUC value than the spaced group. The highest difference was found between patients without pleural effusion and with severe pleural effusion (AUC = 0.99). Poor distinction was noted between mild and moderate cases (AUC = 0.76). The evaluation results of the validation cohort are shown in Fig. 6c and d, which exhibited the same trend as Fig. 6a and b.

### Confusion matrix analysis

**Fig. 7:**
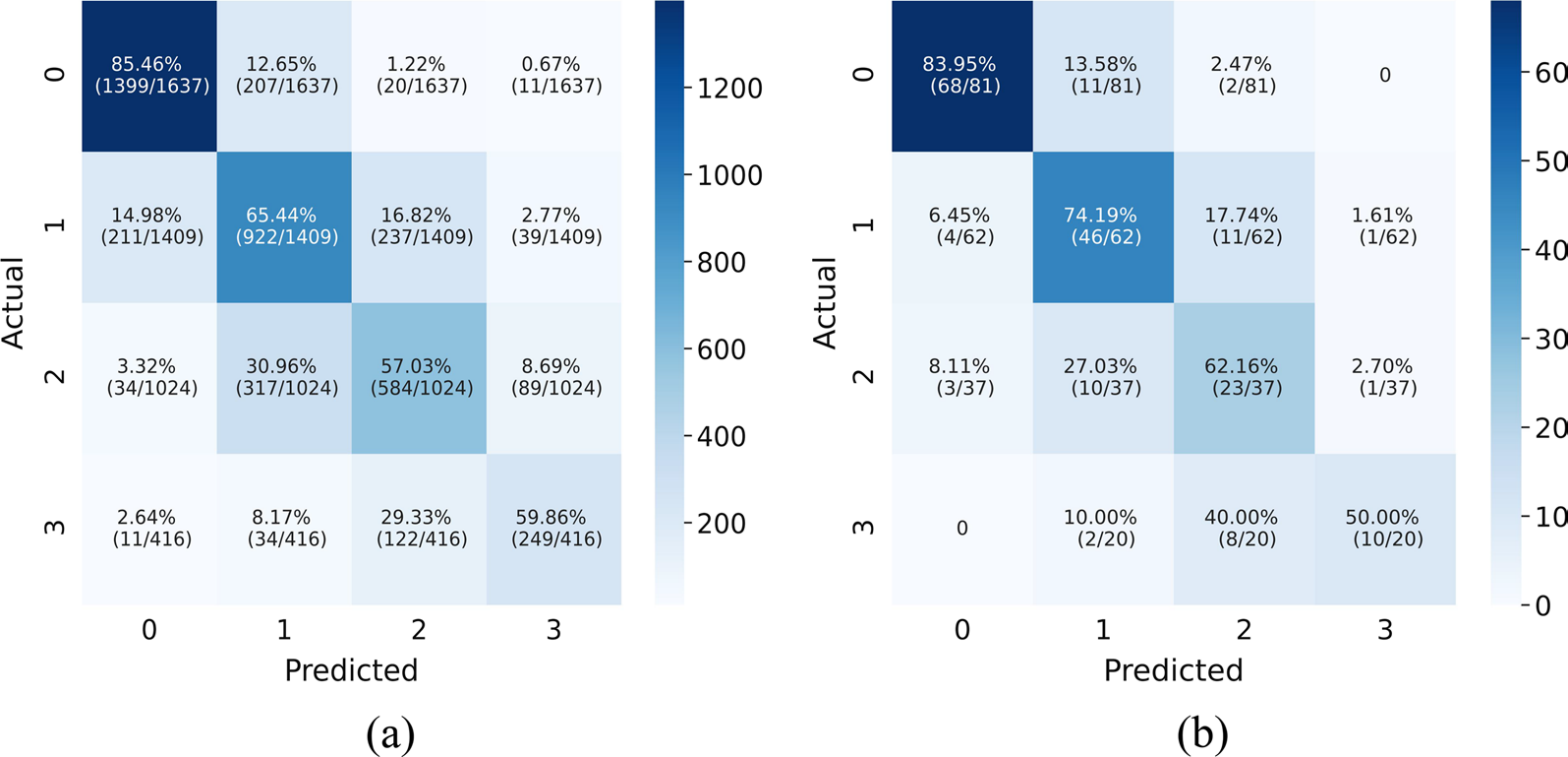
Confusion matrices from the testing cohort and validation cohort. The percentage indicates the proportion of the correct result of the prediction in the actual mark of the current category. The number of correct predictions and markers in the current category is shown in parentheses. **a** Confusion matrices from the testing cohort. **b** Confusion matrices from the validation cohort

The confusion matrix results of the model on the testing and validation cohorts were calculated and shown in Fig. 7. The testing cohort was distributed in a 4*4 matrix according to the labeled labels and the predicted results. Each square represents the ratio of the predicted severity level to the actual severity level. Total data volume and prediction are shown for each level. The results showed that the prediction accuracy of chest radiographs without pleural effusion was 85.46%. Among the 14.54% of the prediction errors, 12.56% were predicted to be mild pleural effusion, and only 1.89% were predicted to be moderate or above. The prediction accuracy rates of small, moderate, and large effusion were 65.44%, 57.03%, and 59.86%, respectively. The classification of prediction errors is basically in the adjacent degree category. Figure 7b shows the results of data verification marked by the doctor. The accuracy rates of no pleural effusion, small effusion, moderate effusion, and large effusion are 83.95%, 74.19%, 62.16%, and 50.00%, respectively. Similar to that in the testing cohort, the classification of prediction error is basically in the adjacent degree category.

### Model interpretation

**Fig. 8:**
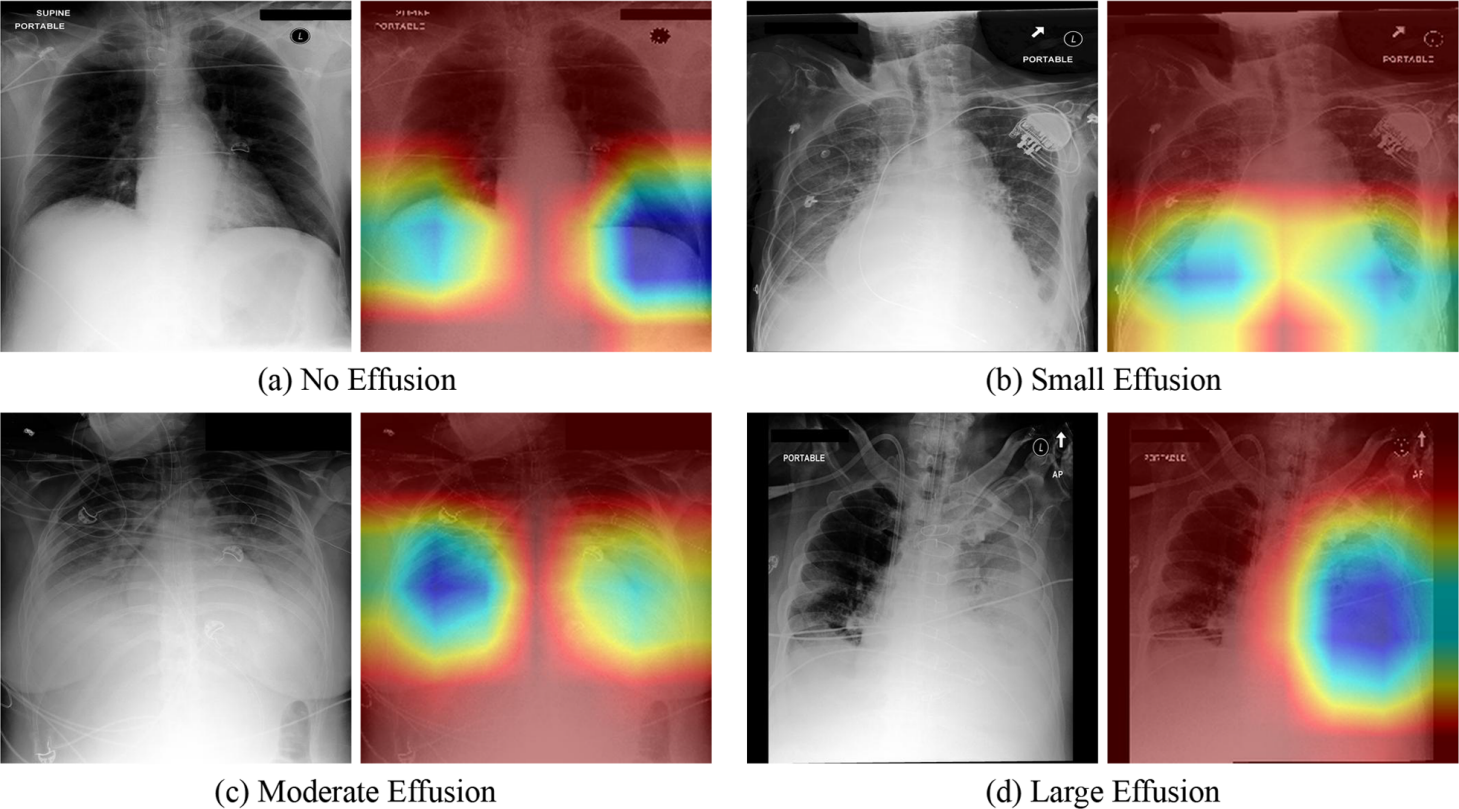
Grad-CAM heatmaps that highlight important regions for the model prediction and its source X-ray chest radiograph

Grad-CAM [40] can visualize the main areas predicted by the model on X-ray chest radiographs and thus was used to calculate the heat map of the last convolutional layer of the model and superimpose it on the original image. Figure 8 shows the comparison result of the original image and the heat map superimposed on the four severity levels. The red part that gathers inward to the blue part is active, indicating that the model pays particular attention to this area.

## Discussion

Although the clinical definition of no pleural effusion does not exist, most people have a small amount of pleural effusion acting as a lubrication during breathing exercises. This work mainly focuses on patients with COPD. A normal chest radiograph is defined as no pleural effusion, and the costophrenic angle is clearly visible [41]. Figure 2 shows the statistical results for different degrees of effusion data. The number of chest radiographs is inversely proportional to the effusion severity, and this finding is in line with the objective situation.

Grading pleural effusion severity is an extension of previous research. Deep learning can identify pleural effusions and other pathological conditions in chest X-rays at a level that surpasses experts [42, 43]. Whether in the clinical diagnosis or when the radiology department gives an X-ray report, the severity of the pathological situation will be judged. To the authors’ knowledge, no research has automatically and quantitatively assessed pleural effusion severity. The parameter tuning result of the NNI tool [28] showed good performance of the network structure model of DenseNet121 [30] with an accuracy rate of 0.730 (loss: Focal, Resample Way: None, LR: 0.005, Optimizer: Adagrad). The input of each layer of the DenseNet structure comes from the output of all previous layers. This process reduces the disappearance of gradients and effectively utilizes the image features. The 121-layer deep structure strengthens the learning ability and obtains better results than other models. Focal Loss [36, 44, 45] solves the model training problem caused by sample imbalance from the perspective of sample difficulty and easy classification. This becomes a classification problem of imbalanced samples, for example, due to the different number of chest radiographs of different severity. The problem caused by sample imbalance is that categories with a small number of samples are more difficult to classify. Therefore, from the perspective of sample classification difficulty, the Focal Loss function focuses on difficult samples, which solves the problem of low classification accuracy for categories with few samples. Of course, difficult samples are not limited to categories with few samples. Focal loss not only solves the problem of sample imbalance, but also helps to improve the overall performance of the model, so it is better than cross entropy loss optimization. According to the training results of NNI, data sampling is not advisable because downsampling reduces the amount of data and the fitting ability of the model. Although oversampling increases the amount of data, the excessive copying of the same sample does not enhance the learning ability of the model. After Grad-CAM [40] extracted the activation state of the last convolutional layer, the model locates the key areas that must be thoroughly observed. Therefore, the model serves as the basis for obtaining the prediction results.

ROC curve evaluation was used for the prediction results of DenseNet121. The severity label was extracted from the radiology report of MIMIC-CXR. Other chest radiograph datasets, such as Chexpert [18], NIHChest-Xray [46], do not provide original radiology reports or similar severity labels. Hence, the predictive ability of the proposed model on other datasets cannot be verified. Although ‘no pleural effusiono’ classify with the other three categories is essentially two classifications, similar to whether pleural effusion can be detected on a chest radiograph. However, simple comparisons are essentially undesirable because of the difference on data distribution and model function goals. Hence, 30% of the dataset was used to test the performance of the model. Additional 200 cases were randomly selected and marked by the clinician as an additional verification result to prove the superior performance of the model.

In Fig. 6, the macro average calculates the indicators of each class independently and then takes the mean value to equally treat all classes; the micro average aggregates the contributions of all classes to calculate the average indicator [47]. Similarities in the macro and micro results for the test and validation sets show that the model effectively solves the problem of data imbalance, which is common in medical datasets. A model that can effectively solve the imbalance problem is of great help to its promotion [48]. In the distinction between single category and the other three categories, the discrepancy between small pleural effusion on the testing cohort and the other three categories was not evident. On the basis of the ROC curve of the pairwise comparison, the main reason is the unclear distinction between levels 1 and 2. According to the corresponding radiology report, the language expression of these patients was relatively unclear. The main reason is that some expressions such as ‘left small, right moderate,’ ‘moderate relaxation,’ and other sentence patterns affected the labeling results. The secondary reason is that the visual discrimination between levels 1 and 2 is not as good as that between other levels, thus further affecting the training of the model as indicated by the validation cohort results.

The predicted results and labels are summarized by category to obtain a confusion matrix [49] for further evaluation. The confusion matrix shows the prediction results of each sample. The model distinguishes non-adjacent categories well, but the accuracy needs to be improved when differentiating adjacent categories. The results had similar performance whether the labels are based on keyword extraction or are manually annotated. The main reason for this result is that the severity of the definition of pleural effusion is determined by the size and height range of the shadow on the chest X-ray [50]. Sometimes it is difficult to clearly define whether the amount of pleural effusion is above or below the reference point. But what is certain is that the model constructed in this article can effectively distinguish chest radiographs of different severity. And from this, it can be inferred that chest radiographs that are incorrectly judged as adjacent categories are likely to be data with small visual differences and blurred boundaries.

The proposed method has its limitations. First, this work only used frontal chest radiographs and did not distinguish between left and right chest cavities. Accurate positioning will be of great help to clinical diagnosis. Second, the constructed model faces difficulty in stratifying data with fuzzy boundaries. If a stepless severity score can be developed, then an accurate diagnosis can be made. Third, only the most direct transfer learning model was validated. Many different learning modes are available in the field of deep learning, such as semi-supervised learning, small-sample learning, and reinforcement learning. Each has its own advantages. Exploring a model that is suitable for grading the severity of pleural effusion will be the focus of future research.

## Conclusions

The proposed model for the assessment of pleural effusion severity can be used to upgrade or downgrade care and to monitor the efficacy of treatment, especially in the ICUs. This model can classify the pleural effusion grades on chest radiographs, thus allowing clinicians to compare CXR images using quantitative and objective measurements.

## Data Availability

The MIMIC-CXR data were available on the project website at https://www.physionet.org/content/mimic-cxr/2.0.0/. The MIMIC-CXR-JPG data were available on the project website at https://www.physionet.org/content/mimic-cxr-jpg/2.0.0/.

## Abbreviations

COPD: Chronic obstructive pulmonary disease
MIMIC-CXR: Medical information mart for intensive care chest X-ray
NNI: Neural Network Intelligence
ROC: Receiver operating characteristic
AUC: Area under the curve
ICUs: Intensive care units
